# Acneiform eruption associated with Janus kinase inhibitors in pediatric inflammatory bowel disease: Case series and clinical management guidance

**DOI:** 10.1002/jpr3.70255

**Published:** 2026-10-12

**Authors:** Tina H. Tran, Daniel Park, Regina‐Celeste Ahmad, Malika Goel, Sofia Verstraete, Sabina Ali

**Affiliations:** ^1^ Department of Pediatrics UCSF Benioff Children's Hospitals Oakland Oakland California USA; ^2^ Department of Dermatology, Division of Pediatric Dermatology UCSF and UCSF Benioff Children's Hospitals Oakland California USA; ^3^ Department of Pediatrics, Division of Pediatric Gastroenterology Hepatology and Nutrition UCSF Benioff Children's Hospitals San Francisco California USA

**Keywords:** acne, dermatology, JAKi side effects, patient counseling

## Abstract

Janus kinase inhibitors (JAKi) are increasingly used for refractory inflammatory bowel disease (IBD) in pediatric patients but are associated with significant dermatologic adverse effects. Acneiform eruptions have been reported in up to 30% of adults, with a greater incidence suspected in adolescents. We performed a retrospective review of 66 pediatric IBD patients treated with JAKi at UCSF Benioff Children's Hospitals (2022–2025), including 41 on upadacitinib and 25 on tofacitinib. Eight patients (12.1%) developed new‐onset or worsening moderate to severe acne requiring dermatology referral for management; rates were higher with upadacitinib (6/41, 14.6%) than tofacitinib (2/25, 8.0%). Acne onset was early (median 3.5 weeks) and predominantly inflammatory. Four patients required JAKi discontinuation, and two developed acne fulminans requiring hospitalization. Management often required systemic therapy and multidisciplinary care. These findings highlight the need for anticipatory counseling, early recognition, and prompt dermatologic involvement to reduce morbidity and support continuation of effective IBD therapy.

## Abstract (highlights)

**What is Known**

- Janus kinase inhibitors (JAKi) are associated with an overall acne incidence of 8.6% in patients with inflammatory bowel disease (IBD).
- Often referred to as “JAKne,” these acneiform eruptions typically occur within 2–12 weeks of JAKi initiation.

**What is New**

- Prior acne history may predict dermatologic intolerance to JAKi treatment, supporting early dermatology involvement.
- JAKi‐associated acne may be more inflammatory and treatment‐resistant than typical adolescent acne, with severe cases requiring isotretinoin or JAKi treatment modification.

## 1 INTRODUCTION

A substantial proportion of patients with inflammatory bowel disease (IBD) experience inadequate response or intolerance to first‐ and second‐line therapies.^1^, ^2^ While aminosalicylates, corticosteroids, immunomodulators, and biologic agents are effective for many, a subset of patients develop refractory disease necessitating escalation to advanced therapies, such as Janus kinase inhibitors (JAKi).^3^ JAKi have emerged as an off‐label oral therapeutic option for pediatric patients with moderate to severe IBD and other immune‐mediated conditions. By inhibiting the JAK‐STAT signaling pathway, these small‐molecule agents modulate multiple cytokine receptors implicated intestinal inflammation and offer an alternative to injectable biologics.

Although JAKi are generally well tolerated, dermatologic adverse effects including acneiform eruptions have been increasingly reported in both dermatology and gastroenterology literature, though pediatric data remains limited.^4^, ^5^ Reported rates of acne among patients undergoing JAKi treatment range from 6% to 32% of patients, with incidence in IBD populations as high as 20%, particularly among patients treated with selective JAK1 inhibitors such as upadacitinib.^4^, ^6^, ^7^ Recognition of this adverse effect may be especially challenging in adolescents, in whom baseline acne is already common. As a result, acne associated with JAKi therapy may be overlooked during anticipatory counseling and early treatment discussions. When severe acne develops unexpectedly, it can contribute to psychological distress, social withdrawal, uncertainty regarding the cause, and discontinuation of what could have otherwise been effective therapy.

The pathophysiology of JAKi–associated acne is not fully understood. Proposed mechanisms include altered cytokine signaling affecting keratinocyte differentiation, sebaceous gland activity, and cutaneous immune dysregulation.^8^, ^9^, ^10^ A dose‐dependent relationship has been described, most commonly observed in patients undergoing upadacitinib or tofacitinib treatment.^11^, ^12^, ^13^, ^14^, ^15^ Clinically, these eruptions are often characterized by inflammatory papules and pustules that may resemble or exacerbate inflammatory acne vulgaris. Distinguishing medication‐associated acne from baseline adolescent acne can therefore be challenging, particularly in patients receiving concurrent corticosteroids or other immunomodulatory therapies that may independently contribute to acne development.

In our case series, we describe eight pediatric patients who developed clinically significant acne following initiation of JAKi therapy. The objectives of this report are to characterize JAK inhibitor‐associated acne in pediatric patients with IBD and to offer practical recommendations for anticipatory counseling, timely identification, and coordinated multidisciplinary care to preserve treatment continuity and quality of life.

## 2 METHODS

### 2.1 Ethics statement

This study was approved by the institutional review board at the University of California, San Francisco as study #12‐12137.

### 2.2 Data collection

We conducted a retrospective review of pediatric patients treated at the University of California, San Francisco Benioff Children's Hospital between January 2022 and June 2025 who were initiated on JAKi therapy for refractory IBD. Patients were included in this case series if they developed new‐onset acne or acute worsening of acne following JAKi initiation requiring referral to dermatology for management. Acne severity was dermatologist‐defined; no standardized grading scale was uniformly applied due to the retrospective design.

Clinical data were extracted from the electronic medical record and included patient demographics, prior biologics, type and dosage of JAKi, steroid exposure, timing of acne onset, acne severity based on dermatology assessment, acne‐directed treatments, and the impact of dermatologic symptoms on medication adherence. Patients were followed from the date of JAKi initiation through May 2026.

### 2.3 Statistical analysis

Qualitative descriptors were used to summarize patient demographics and clinical characteristics. Categorical variables were presented as frequencies and percentages, while continuous variables were reported as medians with interquartile ranges (IQRs).

## 3 RESULTS

During the study period, 66 pediatric patients in our IBD registry received JAKi therapy for refractory disease, including 41 treated with upadacitinib and 25 with tofacitinib. Eight patients (12.1%) developed clinically significant acne following JAKi initiation (Table 1). The cohort was evenly distributed by sex and disease subtype, with four patients each with Crohn's disease and ulcerative colitis and two males and two females within each subtype. Five patients had a prior history of acne. The median age at acne onset or acute worsening was 17 years (IQR 16–18.5; range 14–21 years). Acne occurred in 6 of 41 patients treated with upadacitinib (14.6%) and 2 of 25 treated with tofacitinib (8.0%).

**Table 1:** Demographics, treatment, and outcomes of patients with acneiform eruption associated with JAKi initiation.

Case no	Sex/Age	Disease	Prior acne history	Inciting JAKi	Time to acne onset/flare (weeks)	Acne subtypes (severity)	Failed acne treatment	Current acne treatment	JAKi dose change	Time to resolution (weeks)
1	M/17	Crohn's disease	No	Tofacitinib	2	Acne vulgaris (mild to moderate)	Tretinoin 0.025%/0.05%/0.1%, Benzoyl peroxide 5%	Benzoyl peroxide 10%, Tazorac 0.05% Clindamycin topical 3x week	No change	100
2	M/16	Crohn's disease	No	Upadacitinib	12	Acne vulgaris (mild to moderate)	N/A	Tretinoin 0.025%	No change	Improved, not yet resolved at 24 weeks
3	F/17	Crohn's disease	Yes	Upadacitinib	3	Acneiform eruption (moderate)	N/A	Topical Ketoconazole 5x week	No change	Not improved or resolved at 48 weeks
4	F/17	Crohn's disease	No	Upadacitinib	2	Acneiform eruption (unknown)	N/A	Adapalene 0.3% gel, Tacrolimus 0.1% ointment, Sulfur 10% Cleanser	45–30 mg	Resolved after completing 12 weeks of induction dosing at 45 mg
5	M/14	Ulcerative colitis	Yes	Upadacitinib	4	Acne vulgaris progressed to cystic acne/acne fulminans	Salicylic acid wash, Tretinoin 0.1%, Intralesional triamcinolone, Prednisone, Bactrim, Azithromycin	Isotretinoin	Discontinued Upadacitinib after development of Acne Fulminans at 8 weeks	30
6	M/20	Ulcerative colitis	Yes	Upadacitinib	2.5	Acne vulgaris/acneiform eruption (moderate)	Topical Clindamycin 1%	Tretinoin 0.025%, Benzoyl Peroxide 5% wash 2x week	Discontinued Upadacitinib 3 weeks after initiation due to acne burden	11
7	F/16	Ulcerative colitis	Yes	Tofacitinib	4	Acne vulgaris progressed to cystic acne/acne fulminans	Spironolactone 50 mg, Adapalene 0.1% Cream, Tretinoin 0.025%/0.05%, Intralesional Triamcinolone	Combined oral contraceptives, Tretinoin 0.1%, Benzoyl peroxide 5% wash, Spironolactone 150 mg	Discontinued Tofacitinib at 20 weeks due to acne burden	31.5
8	F/21	Ulcerative colitis	Yes, with isotretinoin treatment 5 years prior	Upadacitinib	4	Cystic acne (unknown)	Spironolactone 100 mg, Benzoyl Peroxide 5% wash, Topical Clindamycin 1% BID	Spironolactone 100 mg, Hibaclens and Benzoyl Peroxide 5% wash, Topical Clindamycin 1% BID	Discontinued Upadacitinib after 32 weeks due to frequent upper respiratory tract infections and acne burden.	49

Abbreviations: F, female; JAKi, Janus kinase inhibitors; M, male.

Onset or acute flare of acne occurred early following JAKi initiation, with a median time to onset of 3.5 weeks (IQR 2.4–4.0; range 2–12 weeks). All patients developed acne severe enough to require dermatology referral, with predominantly inflammatory morphologies including papules, pustules, and nodules. Two patients progressed to severe cystic acne and acne fulminans requiring hospitalization for pain control and intensive dermatologic management (Cases 5 and 7). Acneiform eruptions did not follow a typical “T zone” distribution, but involved the whole face in all patients, with truncal involvement of the back, chest, and shoulders observed in four out of the eight patients.

Management strategies varied and included topical therapies such as benzoyl peroxide washes, topical retinoids, topical antibiotics, and topical antifungal agents, as well as systemic treatments including oral antibiotics, spironolactone, systemic corticosteroids, and isotretinoin. Despite standard acne therapies, the disease course was frequently prolonged and recalcitrant. Five patients required JAKi dose reduction or discontinuation due to acne severity or intolerance. Acne resolution was achieved in six of eight patients, with a median time to resolution of 30.8 weeks (IQR 12.0–49.0; range 11–100 weeks). Four of these patients discontinued JAKi due to acne intolerance, one patient (Case 1) reached acne resolution with topical medications, and one patient (Case 4) had resolution of symptoms after completing the induction phase. The other two patients who continued JAKi therapy (Cases 2 and 3) had incomplete resolution at last follow‐up, despite ongoing dermatologic treatment.

## 4 DISCUSSION

In this case series, we describe eight pediatric patients with IBD who developed acne requiring dermatology referral after initiation of JAKi therapy, with onset typically occurring in the first several weeks of treatment. Although acneiform eruptions associated with JAKi have been increasingly reported in dermatology and rheumatology literature, they remain underrecognized within pediatric gastroenterology. As a result, this adverse effect may not be adequately addressed during anticipatory counseling. Our findings highlight the clinical and psychosocial burden of JAKi‐associated acne and emphasizes the need for anticipatory counseling, early recognition, and structured management strategies when initiating JAKi therapy in adolescents. To support clinical practice, we propose a standardized counseling script for use prior to JAKi initiation that providers can incorporate within their own patient education materials or after visit summaries (File S1).

Many pediatric IBD patients receiving JAKi have complex treatment histories with frequent exposure to systemic corticosteroids, which are known to exacerbate acne through increased sebaceous gland activity and alterations in skin microbiota. However, patients in our cohort had repeated steroid courses without developing clinically significant acne until starting JAKi therapy. The close temporal relationship between JAKi exposure and acne onset, coupled with the improvement seen after dose reduction or discontinuation in several cases, suggests that JAKi therapy is the primary driver of acne in this population, with corticosteroids potentially serving as a secondary contributing factor. This interpretation is supported by a recent multicenter cohort study by Honap et al., who found no significant increase in acne among patients receiving concomitant corticosteroids and JAKi therapy compared with those receiving JAKi therapy alone.^16^ Together, these findings suggest that while corticosteroid exposure may contribute to acne severity in susceptible individuals, JAK inhibition appears to be the predominant factor associated with acne development.

Adolescent physiology may further amplify the risk of JAK inhibitor–associated acne. Elevated androgen levels in adolescent patients stimulates sebaceous gland activity and sebum production, creating a lipid‐rich environment that promotes *Cutibacterium acnes* proliferation and downstream inflammation.^17^ This baseline susceptibility may predispose pediatric patients to more severe or treatment‐resistant acne in the setting of JAKi therapy. Consistent with this hypothesis, four out of five patients in our cohort with a history of acne had substantial flares that led to treatment discontinuation. Similar findings have been reported by Honap et al., who found that a history of acne vulgaris was associated with an approximately 4.9‐fold increased risk of developing severe JAK inhibitor–associated acne.^16^ Recognizing these overlapping factors is critical for accurate attribution, anticipatory counseling, and therapeutic decision‐making.

The pathophysiology of JAKi‐associated acne is incompletely understood but may involve dysregulation of cytokine signaling pathways that govern follicular keratinization, sebaceous gland function, and cutaneous immune homeostasis. Inhibition of pathways involving interleukin (IL)‐6, IL‐23, and interferon signaling may contribute to an inflammatory acne phenotype distinct from classic acne vulgaris.^18^ In our series, acne was predominantly inflammatory, with two patients progressing to acne fulminans requiring hospitalization for pain control and intravenous antibiotics, underscoring the potential severity of this adverse effect. Upadacitinib, a selective JAK1 inhibitor, was more frequently implicated than tofacitinib, a pan‐JAK inhibitor, consistent with prior cohort studies reporting a stronger association between acneiform eruptions and JAK1 selective inhibition.^11^, ^12^, ^13^, ^14^, ^15^ This difference may reflect the distinct roles of JAK isoforms in cutaneous inflammation and acne pathogenesis. JAK1 mediates signaling through cytokine pathways involved in immune and inflammatory regulation, whereas JAK2 primarily mediates hematopoietic signaling.^19^ Notably, one study found overexpression of JAK1 and JAK3 in acne lesions, where JAK2 expression did not differ between acne vulgaris lesions and healthy skin.^10^ A systematic review including all randomized clinical trials of JAKi reported the highest incidence of acne with JAK1 inhibitors and no statistically significant association with pan‐JAK inhibitors.^9^ Together, these findings may support the hypothesis that selective JAK1 inhibition may confer a greater risk of acneiform eruption, although further studies are needed to clarify the underlying mechanism.

Adolescence represents a particularly vulnerable period during which visible dermatologic changes can significantly impact self‐image, social functioning, and mental health. In our cohort, acne contributed to decreased medication adherence and led to dose reduction or discontinuation of JAKi therapy in most of our patients. Although all cases were severe enough to prompt dermatology referral, the true prevalence of JAKi‐associated acne in pediatric patients is likely underestimated, as cases may be dismissed as idiopathic acne common in this age group, especially if symptoms are mild. These factors, combined with the retrospective nature of this study, highlight the need for prospective surveillance and standardized documentation of dermatologic adverse events.

Management of acne in the setting of JAKi therapy generally follows standard acne treatment principles, including topical retinoids, benzoyl peroxide, and systemic therapies when indicated. However, in our experience and alignment with existing dermatologic literature, JAKi‐related acne tends to be more inflammatory and recalcitrant, frequently requiring escalation to systemic isotretinoin or modification of IBD therapy. Early dermatologic involvement facilitated timely escalation of care and helped avoid prolonged morbidity. Importantly, interdisciplinary collaboration between gastroenterology and dermatology allowed for individualized risk‐benefit discussions and, in some cases, continuation of effective IBD therapy with concurrent acne management. To facilitate early recognition, counseling, and standardized initial management, we propose a practical education and treatment algorithm for pediatric patients initiating JAKi therapy for IBD. This includes pre‐treatment referral to dermatology for patients with preexisting moderate to severe acne, given that they are increased risk of developing severe JAKi‐associated acne and directed therapies can take months to achieve full effect (Figure 1).

**Figure 1:**
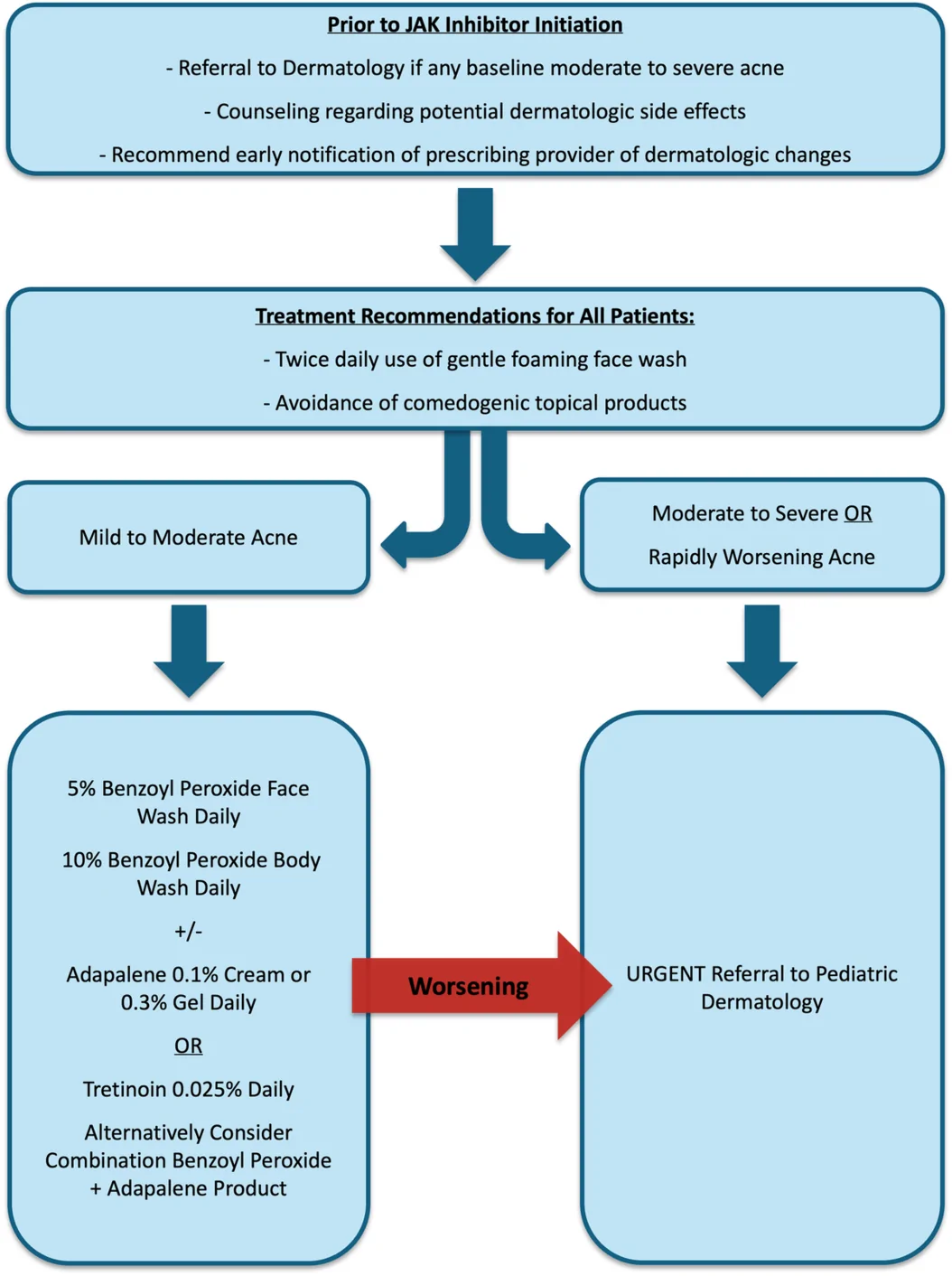
Proposed patient counseling prior to initiation of JAK inhibitor and treatment algorithm for prevention and management of acneiform eruption. JAK, Janus kinase.

Beyond clinical severity, JAKi‐associated acne has substantial implications for healthcare utilization and quality of life. Additional specialty visits, medication costs, missed school days, and psychological distress contributed to the overall burden of care for these patients and families. Our findings reinforce those dermatologic adverse effects, though often perceived as minor, can have outsized effects on adherence, satisfaction, and long‐term outcomes. Incorporating routine skin assessments and proactive counseling into baseline care when initiating JAKi for IBD represents a practical opportunity to mitigate these downstream effects.

## 5 CONCLUSION

This case series has limitations, including its retrospective design, small sample size, and single‐center setting. Nevertheless, it contributes to the limited pediatric gastroenterology literature by characterizing the clinical course, severity, and management challenges of JAK inhibitor‐associated acne in adolescents with IBD. Until larger prospective studies are available, the counseling script and management algorithm proposed here offer a practical framework to enhance early recognition, prompt referral, and shared decision‐making. Greater awareness of this adverse effect will better prepare patients and families, support adherence to effective therapies, and promote more comprehensive, patient‐centered care.

## CONFLICT OF INTEREST STATEMENT

The authors declare no conflicts of interest.

## Supporting information


**Supplementary Material 1.** Recommended standardized counseling script for patients starting JAKi therapy.

## Data Availability

The data that support the findings of this study are available from the corresponding author upon reasonable request.
