# Supplementary material for: Acneiform eruption associated with Janus kinase inhibitors in pediatric inflammatory bowel disease: Case series and clinical management guidance
Source: JPGN Rep. 2026 Oct 12:10.1002/jpr3.70255. Online ahead of print. doi: 10.1002/jpr3.70255 (PMC13656293; doi:10.1002/jpr3.70255)
Supplement: Supplementary file 1 [file JPR3-9999-0-s001.docx]

Information About Skin Changes While Taking JAK Inhibitors
Approximately 30% of patients who start treatments with JAK inhibitors notice new or worsening acne, usually within the first four weeks of treatment. While this side effect can be distressing, it is usually manageable and can improve with supportive care. Please let your prescribing provider know early if you notice any new skin changes, acne, or rashes so we can intervene promptly and avoid interruption of therapy.

General Skin Care Recommendations
To help prevent or minimize skin irritation, wash your face twice daily (morning and evening) with a gentle foaming cleanser. Avoid using heavy or comedogenic (pore-clogging) products such as oily make-up, thick creams, etc.

When to Contact Your Provider
Contact your gastroenterology team promptly if acne becomes painful, widespread, or begins to affect your daily comfort. Early recognition and treatment can prevent worsening and help you stay on your JAK inhibitor safely and comfortably.
